# Stimulus-Response Conflict Tasks and Their Use in Clinical Psychology

**DOI:** 10.3390/ijerph182010657

**Published:** 2021-10-12

**Authors:** Thomas Kleinsorge

**Affiliations:** Leibniz Research Centre for Working Environment and Human Factors (IfADo), Ardeystrasse 67, D-44139 Dortmund, Germany; kleinsorge@ifado.de

**Keywords:** conflict, conflict tasks, clinical psychology, cognitive psychology

## Abstract

This article reviews the historical usage of the concept of ‘conflict’ in psychology and delineates the design and development of three basic conflict tasks (Stroop, Flanker, Stop Signal). Afterwards, important theoretical concepts to account for conflict processing are introduced. In the second part, the usage of these tasks in clinical psychology is considered. The article closes with some reflections regarding factors that may have been hitherto largely neglected in this respect.

## 1. Introduction

### 1.1. Overview

The outline of the present article is as follows. Beginning with a brief introduction on the way the concept of ‘conflict’ has been considered in the history of psychology, three prominent experimental paradigms for introducing conflict will be described. In these paradigms, conflict is induced by the presentation of stimuli which, beyond being to-be processed in a way that conforms with instructions, induce other cognitive processes that interfere with this instruction-based information processing. Although these conflict tasks were originally designed to induce conflict on a specific level of information processing (e.g., attentional, response-related), it turned out that these tasks may induce conflict on several levels simultaneously, which poses some interpretational challenges. Afterwards, relevant theoretical concepts will be introduced. Then, the use of variants of these tasks in clinical psychology is reviewed. This use is most often based on ‘clinical’ versions of these conflict tasks that are supposed to be sensitive to pathological alterations of information processing; they are revealed by comparing the performance of a clinical group with a control group, or by tracing the effects of therapy in terms of an improved task performance that makes performance more similar to the performance of non-pathological participants. However, the transfer of these conflict tasks into a clinical context is often accompanied by structural changes of the tasks that alter their underlying processing requirements, sometimes turning them into completely different tasks, despite preserving superficial similarity with their original prototypes. The article closes with some reflections regarding possible mechanisms underlying certain patterns of performance of these tasks, as exhibited by clinical populations.

### 1.2. Conflict in Psychology

In one way or another, the notion of conflict has pervaded almost all branches of modern psychology since the beginning of the twentieth century. One could even argue that the resolution of conflict provides a conceptual engine that transforms unobservable entities (such as drives, response tendencies, but also personality, goals, and so on) into observable behavior. For example, in psychoanalytic theory, the Ego, which brings psychological forces into contact with reality, is thought to arbitrate conflicts between biological drives (the Id) and cultural norms (the Super-Ego). Ach [1] was the first to design an experimental paradigm for inducing conflict by first associating certain stimuli with particular responses, which in a later phase of the experiment had to be overcome by replacing the earlier responses by newly learned ones. In a somewhat similar vein, behavioristic psychologists used the notion of conflict among several conditioned or unconditioned responses to account for complex overt behavior (e.g., ‘conflict theories’ of ‘experimental neurosis’, cf. [2].

One of the most influential accounts of conflict in the first half of the twentieth century is Lewin’s typology of motivational conflicts [3]. Lewin distinguished between three basic types of conflict (approach-approach, avoidance-avoidance, approach-avoidance) as well as double approach-avoidance conflicts, which are basically conflicts between two alternatives that incur in both approach-avoidance conflicts. Lewin’s work as well as subsequent work by Miller [4], regarding the steepness of avoidance- and approach-gradients, has spawned a tremendous amount of work, including the influential Behavioral Inhibition System theory by Gray [5], that has been applied to a number of clinical problems, such as anxiety (e.g., [6]).

With the advent of cognitive psychology, the notion of conflict was underlain with metaphors from information technology (e.g., interference, cross-talk) that provided this concept with a more mechanistic flavor. Furthermore, a number of experimental paradigms emerged that implemented conflict in a rather controlled manner. In line with the early selection accounts of attention that dominated the early phase of cognitive psychology, the first experimental paradigms conceived of conflict primarily as a conflict among different sources of information (e.g., dichotic listening). With late selection accounts gaining ground, conflict was increasingly located at the response side. Nowadays, it has been shown that even superficially simple conflict tasks (which were initially thought to capture conflict either on the stimulus or response side) can induce conflicts on a number of levels simultaneously. While this development has led to a dramatic increase of knowledge regarding human information processing during the past few decades, it also identified a number of problems and pitfalls associated with employing experimental conflict tasks at face value. Furthermore, it is now well established that tasks are represented on several levels that are insufficiently characterized by the traditional distinction between a perceptual, central, and motor level. As a concomitant, the number of potential conflicts has increased accordingly.

As will be shown in detail below, even very simple tasks may engender a number of processes that can lead to a multitude of conflicts. Even worse, simplifying tasks even further provides no guarantee that the conflicts they induce become more transparent. To the contrary, one may find oneself in a situation in which, for example, restricting the set of stimuli presented within a task has the potential to pave the way to induce new types of conflict that could be neglected with a larger stimulus set. For example, a task commonly used to induce conflict is the Eriksen flanker task, where participants have to respond to a central target while ignoring distractors that flank the target. Conflict adaptation in this task is signified by more efficient performance to a target with incongruent distractors if the current trial is preceded by another incongruent trial, as compared to a preceding congruent trial. Bugg [7] showed that the conflict adaptation effect in the flanker task is masked by additional and counteracting negative priming (a form of inhibition of a previously ignored stimulus attribute), with a smaller as compared to a larger stimulus set, implicating that a more complex task allows for a purer measure of conflict adaptation in comparison to a less complex task.

## 2. Inducing Conflict: Basic Conflict Tasks

In the following, three types of stimulus-response conflict tasks are introduced that have in common a relevant stimulus-response, which maps the implementation and is subject to interference by a nominally irrelevant stimulus or stimulus attribute. In the ‘classical’ versions of these tasks, interference is due to irrelevant stimulus attributes being located on the same (or a conceptually closely related) conceptual dimension as the relevant stimulus attribute, but corresponding to a competing response (e.g., the meaning of a color word whose color is to be categorized, or stimuli that surround a target of the same type). In the influential taxonomy of Kornblum, Hasbroucq, and Osman [7], these are ensembles of the either type 2, 4a, or 4b. In clinical versions of these tasks, to be discussed afterwards, nominally irrelevant stimulus attributes draw their potential to interfere with the relevant stimulus-response mapping from other sources (to be discussed below) that may change their position in the taxonomy of [8] Explicitly not considered in this section, are tasks that primarily create conflict on the basis of evaluative responses, such as the Implicit Association Test [9], affective priming tasks, or approach-avoidance tasks (cf. [10]). The reason for this restriction is that the focus on evaluations built into the architecture of these tasks introduces additional processes (which is also the case with affective variants of the Simon task, cf. [11]). Furthermore, these paradigms tap into intricate questions regarding the implicitness of measurement procedures and measurement outcomes [12]. These conceptual issues are clearly beyond the scope of this article.

### 2.1. The Stroop Task

The oldest but probably still the most widely used conflict task in experimental psychology is the Stroop task. In its original form [13], this task comprised of four conditions, which were delivered via stimulus cards. In one condition, participants were asked to read out aloud color words that were printed in incongruent colors (e.g., the word red printed in green), whereas the corresponding control condition required participants to read out the same words printed in black (with ‘black’ not occurring as a to-be read color word in either condition). In another condition, participants were asked to name the ink color of words denoting incongruent colors (basically the same stimuli as in the first condition), whereas the corresponding control condition required participants to name the ink color of patches. Stroop’s work followed up earlier work comparing the latencies of reading color words and naming the color of non-word stimuli [14,15], but Stroop’s ingenious move consisted of combining words and colors into two-dimensional stimuli that could be responded to according to different instructions of word-reading and color-naming. The classical observation with these stimuli was that whereas word reading was not compromised by incongruent ink colors (as compared to reading color words printed in black), color naming was severely hampered by incongruent color words (as compared to naming the ink color of patches).

Nowadays, a multitude of two-dimensional (and possibly conflicting) stimuli are known to elicit (at least superficially) similar types of interference, which are often credited to Stroop by the term ‘Stroop-like interference’. For a long time, and following the earlier work of Cattell [14], the original Stroop-interference was customarily attributed to differential practice that made word reading ‘automatic’ whereas color-naming was considered as ‘voluntary’. While this account became increasingly debated from the 1960s and onwards [16], the automatic-voluntary distinction still figures as the perhaps most prominent shorthand account of Stroop-like interference phenomena. This is the case even though the automatic-voluntary distinction itself became heavily disputed in the 1980s [17] and since then has lost almost all credibility as an explanatory concept in cognitive psychology (at least when considered in terms of a dichotomy).

Recent research has shown that Stroop-like interference not only cannot be boiled down to two conflicting processes (one automatic, one voluntary), but is composed of at least three different types of conflict: semantic conflict, task conflict, and response conflict [18]. To complicate matters even further, these conflicts are subject to different forms of cognitive control (e.g., proactive and reactive, cf. [19]). Thus, the universe of Stroop-like tasks spans a wide range of both conflicts and control mechanisms that demand careful task analyses to establish which particular conflict-control configuration a certain experimental protocol sets into operation.

### 2.2. The Eriksen Flanker Task

The flanker task was introduced by Eriksen and Eriksen [20] as a method used to investigate the selectivity of attentional processing. In its original version, the task was to respond to a centrally presented letter that was flanked by three identical noise letters to the left and right. Responses consisted of pressing a left or right lever, with two letters assigned to each response (H and K vs. F and C). Apart from some other control conditions that are now usually omitted, there were three major experimental conditions: the noise letters (flankers) could either be identical to the central target letter, differ from the target letter but belong to the same response set (compatible condition), or belong to the other response set (incompatible condition). The major observation consisted of a pronounced slowing of responses in the latter as compared to the two former conditions, an effect that was termed ‘flanker interference’ later on. Although processes related to the spatial distribution of attention play some role in this respect [21], there is consensus that flanker interference is mainly due to response competition, as it has been shown that the incompatible flanker condition results in a (mostly transient) activation of the incorrect alternative response [22].

There exists a multitude of variants of the original flanker paradigm that differ, for example, with respect to the number and type of stimuli, the spatial layout of targets and distractors, and the (a)synchronicity of target and flanker presentation. What most variants have in common, and which is different from Stroop-like tasks, is that flankers and targets are processed within the same task set: Whereas in Stroop-like tasks, the relevant and irrelevant stimulus attributes are usually processed differently (e.g., categorization of colors vs. reading of words, giving rise to conflict on the task-set level), targets and flankers are of the same type and subject to the same encoding operations in the flanker task. Therefore, there is not much room for task conflict in the flanker paradigm. However, this feature gets lost in variants of the flanker task that employ flanker stimuli that are only semantically related to the targets but of a different type [23].

### 2.3. The Stop-Signal Task

Although the stop-signal task is not a typical stimulus-response conflict task, it induces conflict between a go- and a stop-signal that can be pitted against each other in a methodologically elegant way that has yielded important conceptual insight into the nature of ‘automaticity’, discussed in detail below. Therefore, it is included here.

The first use of a stop-signal task dates back to the 1940s, when Vince [24] devised a task that required participants to track displacements of a horizontal line with a pointer. On some trials, a displacement in one direction was followed by a displacement in the opposite direction with inter-stimulus intervals (ISIs) ranging from 50 to 1600 ms, with shorter intervals meaning that participants had to stop a movement in the direction of the first displacement and perform an opposite movement. Vince observed that with ISIs of 500 ms or shorter, responses to the second stimulus were delayed more than expected, based on individual response times to the first stimulus. He interpreted this observation as indicating that after the presentation of the first stimulus, “the sensori-motor system is refractory to similar stimuli”, which causes an additional delay with ISIs of 500 ms or less. Note that in this account neither conflict nor the concept of inhibition play any role.

About 20 years later, Lappin and Eriksen [25] published an experiment that already included most elements of contemporary stop-signal tasks, including the notion of ‘response inhibition’. Participants were asked to respond to the onset of a light but only if this was not followed by the onset of a second light with an ISI of 0, 12, 33, or 63 ms. The first light was denoted as a “go signal” and the second one as a “stop signal”, with the processing of the two signals considered as “competitive”. The authors observed that the probability of successfully stopping the response to the go signal in case of the presentation of a stop signal was jointly determined by individual mean RT to go trials and the duration of the ISI separating the onset of the go and stop signal in stop trials.

Another 20 years later, Logan and Cowan [17] devised a stop-signal paradigm that can be considered as a blueprint for most stop-signal tasks used nowadays. Participants responded to one of four letters, which were assigned to two responses in terms of a 2:1 mapping. In 25% of trials, this go stimulus was followed by an auditory stop signal with delays ranging from 50 to 500 ms. Based on the assumption of a race between a go and a stop process that were considered as stochastically independent, Logan and Cowan also formulated a mathematical model that allowed for the estimation of the stop-signal reaction time (SSRT) as the latency of an unobservable inhibitory process; the response time to a stop signal cannot be observed directly because there is no overt response.

As SSRT is the main dependent variable of the stop-signal task, and the estimation of SSRT relies on the fulfillment of the basic assumptions of the race model, the stop-signal task is vulnerable to violations of these assumptions. For example, when using a very easy go task with very fast response times, allowing participants to stop their responses necessitates relatively short stop signal delays. This, however, may entail perceptual interference between the processing of go and stop signals, which in turn may violate the assumption that the go- and the stop-process are independent of each other [26]. Furthermore, the probability of stop signals and the distribution of stop-signal delays are prone to affect participants’ expectancies and therefore strategies, which may seriously distort results, a phenomenon that resembles the effects of variable foreperiods [27].

In conjunction with its formal underpinnings, the stop-signal task allows for an empirical determination of degrees of automaticity (as opposed to control) that are otherwise only assumed in many cases. For example, it is widely assumed that ‘automatic’ processes are ballistic (that is, unstoppable). The estimation of SSRT allows one to determine the existence of a ballistic processing component empirically because there should be a flat inhibition function (i.e., no increase of stopping probability as a function of the stop-signal delay) for the duration of the ballistic component (cf. [18], p. 317).

## 3. Conflict: Basic Theoretical Concepts

### 3.1. Dual-Process Accounts of Automaticity and Control

When a participant of an experiment is asked to respond to left-pointing arrows by pressing a right key and to right-pointing arrows by pressing a left key, the arrows activate conflicting response tendencies that go along with a transient activation of the wrong but spatially compatible response. This can be characterized as automatic because it runs counter the intention to press the instructed key and requires the exertion of cognitive control to prevent errors. However, it is quite obvious that the same participant will not, upon seeing a right-pointing arrow outside the laboratory, press (or at least feel the urge to press) any key that happens to be located on its right side while seeing the arrow. This brief sketch alone should cast doubt on the assumption of stimuli being endowed with the power to elicit a response independent of intention, attention, and without being able to stop an eventually activated response tendency—that is, that there is completely stimulus-driven automatic behavior.

However, almost all accounts of interference (and also facilitation) that are induced by conflict tasks refer to the concept of automaticity as opposed to control. Whereas early theories tended to view automaticity mainly as especially fast processing due to extensive practice, automaticity was later considered as a special case of information processing (and behavior) that was not only characterized by its speed but also by its independence from intention and awareness, and its independence from limited attentional resources as well as its ballistic nature (e.g., [28,29]). This ‘monolithic concept of automaticity’ [30] dominated cognitive psychology during the 1970s and 1980s, but was then replaced by different variants of ‘conditional’ or ‘prepared’ automaticity that, apart from dissociating the various features of monolithic automaticity, argued that there is almost no kind of information processing and behavior that is at the same time completely independent of intention, proceeds outside of awareness, and is unstoppable (e.g., [17,31]). Importantly, abandoning the assumption of monolithic automaticity also dismissed the idea that any behavior is either controlled or automatic, as well as the assumption that if a certain behavior is shown to possess one of the features of automaticity, it must possess all of its other features (e.g., that ballistic behavior is unintentional and runs off outside of awareness).

Another problem that is associated with a monolithic concept of automaticity pertains to the fact that almost no observable behavior is process-pure, that is, reflects only one underlying process that may or may not be ‘automatic’ in terms of one or several criteria of automaticity. As discussed with respect to the stop-signal task, overt behavior (or its absence due to successful stopping) may be based on several competing or synergistic processes, which sometimes can only be inferred from overt behavior. One of the most influential approaches to tackle this problem consists of proposing (at least) two routes of information processing that converge upon a single behavior.

### 3.2. Dual-Route Models

The perhaps best-known dual-route model in cognitive psychology is the Dimensional Overlap (DO) model of [8]. The main aim of this model is to account for a large number of stimulus-response compatibility effects in a parsimonious way. The basic tenet of the DO model is that stimulus-response compatibility effects are generally based on dimensional overlap between stimuli and responses. Dimensional overlap is most obvious when stimuli and responses vary on the same dimension (e.g., left and right stimuli are responded to by left and right key presses) but is also present in tasks of the Simon type [32], where participants are asked to respond to a nonspatial attribute of a stimulus that is presented in different spatial locations with a spatial response that varies on the same spatial dimension as the stimulus locations. On the processing level, the DO model posits two routes of information processing, *activation* and *confirmation*. When stimuli and responses vary on the same dimension, a stimulus is assumed to *activate* its corresponding response irrespective of whether this is the correct response according to instructions. In the case of a compatible mapping of stimuli to responses, this activated response is then *confirmed* and executed immediately, whereas it is disconfirmed in the case of an incompatible mapping and has to be transformed or replaced by the appropriate response, resulting in a response slowing in comparison to an immediately executed confirmed response. The DO model assumes the activation process to be automatic in two ways: first, it is assumed that within processing stages (e.g., stimulus identification), recoding and transformation proceed “without any interference or intervention by monitoring and controlling processes” ([8], p. 262). Second, it is assumed that the output of one stage-like stimulus identification is directly transmitted to subsequent stages (e.g., response selection) directly and “without interference and intervention” (ibd.). However, [8] also consider the concept of automaticity as not specified “in sufficient detail with enough confidence about the automatic processes in different tasks to provide much insight at that level” (ibd.). This dual-route structure has been applied to other conflict tasks beyond the area of stimulus-response compatibility in a narrow sense, e.g., conflict tasks of the Stroop and Eriksen types.

### 3.3. Sequential Effects, Conflict Adaptation

Apart from some notable exceptions (e.g., [33,34,35,36]), until the 1990s response-time research in cognitive psychology (including the conflict-related research discussed here) treated single experimental trials as a quasi-natural unit of analysis representative of behavior in general. As pointedly put by Broadbent ([37], p. 876), the general practice was to deliver a stimulus, catch a response, and to ignore what happened before and afterwards. Since that time, there has been a tremendous increase of interest in sequential effects, that is, the dependence of performance in a certain trial on the specifics of its predecessor trials. Of course, sequential effects have always contributed to performance in conflict tasks; they had been neglected, but now became the focus of an enormously growing research interest. Probably the two most prominent approaches in this respect consist of research on task switching and conflict adaptation.

Research on task switching (cf. [38] for review) introduces yet another type of conflict in addition to the types of conflict discussed so far. Simply said, the basic conflict is between continuing what has been done before and switching to a new task. This is akin to the phenomenon of cognitive and/or behavioral perseveration. Although examination of perseveration by means of the Wisconsin Card Sorting Test [39] has a long tradition in neuropsychology, the methodological developments in task switching research have paved the way for much more fine-grained analyses of perseverative behavior.

Probably the most important sequential effect with regard to the performance of conflict tasks is the sequential congruency effect. This effect was first observed with the Eriksen flanker task [40] and consists of the observation that interference effects are reduced after another incongruent trial, as compared to a congruent or neutral predecessor trial. Since then, this effect has been observed with all of the conflict tasks discussed in this article (cf. [41] for review). It has been shown that conflict adaptation is compromised by high trait anxiety [42], whereas the evidence with respect to other pathologies, such as schizophrenia [43] and depression [44], is rather mixed. One reason why there is currently no clear picture regarding the effects of certain pathologies on the conflict adaptation effect is possibly that the effect has become the target of experimental investigations only relatively recently, with its underlying mechanisms still being heavily disputed. These controversies extend to the question inasmuch this effect is really about cognitive control or merely a byproduct of elementary memory processes [45].

The most influential theoretical account of the sequential congruency effect is the conflict-monitoring theory [46], which posits that the detection of conflict induces an adaptive strengthening of task-relevant representations and/or processes that subsequently reduce the effect of conflict. Methodologically, this has led researchers to analyze performance in conflict tasks, not only as a function of the features of the current trial (trial n), but to take (at least) the previous trial (trial n − 1) into account. This has considerably broadened the scope of possible clinically relevant observations in conflict tasks, as one can distinguish between alterations in conflict monitoring (expressing themselves mainly as effects of the characteristics of trial n) and adaptations of cognitive control to the occurrence of conflict (expressing themselves mainly as effects of the characteristics of trial n − 1). One further practical implication of this is that it is necessary not only to control the relative frequency of relevant features of trial n but also to control the frequencies of transitions from trial n − 1 to trial n. Otherwise, one might interpret sequential effects as resulting from features of trial n, as has been done with emotional versions of the Stroop task before [47] could show that most of the ‘emotional Stroop effect’ is a ‘slow’ one, affecting the trial after the presentation of a conflicting emotional stimulus.

## 4. Stimulus-Response Conflict Tasks in Clinical Psychology

In clinical psychology, conflict tasks are often used as a diagnostic tool [48]. One of the major avenues in this respect is to compare a clinical group with a control group on two types of conflict tasks: an affectively neutral task (e.g., the classical Stroop color-naming task with color words as distracters) and a ‘clinical’ version, in which conflicting information is related to the pathology of the clinical group (e.g., an emotional Stroop task, in which distractor words are associated with threat in the case of anxiety as the relevant pathology). Thus, in the clinical version the conflict is not due to an incongruent stimulus activating an incorrect response (saying ‘blue’ instead of ‘green’ in response to the presentation of the word blue in green color) but due to the distracting stimulus feature, compromising the processing of the response-relevant stimulus attribute (e.g., a slowdown of the verbal response ‘green’ to the word ‘sad’ written in green). Along these lines, it has for example been shown that eating-disordered women exhibit more Stroop interference with food- and body-related words as compared to neutral words (cf. [49] for review; cf. also[50]), that depressed participants exhibit larger interference with emotional (positive but more-so negative) words as compared to neutral ones (cf. [51] for meta-analysis; cf. also [52]), or that the attentional control in a classical Stroop task suffers from the concurrent presentation of emotional pictures in highly anxious participants [53]. Regarding the stop-signal task, it has for example been reported that a group with pathological worry proneness made less omission errors and more commission errors when the go signal was a worry-related word and the stop-signal was a neutral word as compared to the reverse condition, an asymmetry that was not shown in a control group [54]. With respect to the flanker task, [55] provided evidence that a stronger tendency for rumination goes along with larger facilitation by negative flankers and smaller interference by positive flankers when the central target is a negative word [56]. With respect to the conflict adaptation effect it has been shown that conflict adaptation is compromised by high trait anxiety [42], whereas the evidence with respect to other pathologies, such as schizophrenia [43] and depression [44], is rather mixed. Apart from diagnostic usages, carefully designed clinical versions of conflict tasks allow for testing hypotheses of the functional locus of these effects, which promises to yield insight into the underlying pathology.

As most clinical studies using conflict tasks have employed some variant of the Stroop task, in what follows this task will serve as the primary example, but most points also apply to the other conflict tasks.

As already mentioned, the development of ‘emotional’ variants of the Stroop task and their transfer into clinical settings goes along with a change of the role that interfering stimulus attributes play. Whereas in the original Stroop task distractor words can be translated into the same response as the color of these words, employing emotional words associated with patients’ personal concerns changes the basic structure of this task. As these distractor words cannot be assumed to activate color words (or associated key presses) as responses, the types of conflict that are assumed to contribute to conventional Stroop interference (semantic conflict, task conflict, and response conflict, cf. [18]) are not plausible candidates as sources of interference. This suggests that other processes are likely to play a key role in this respect.

It has been shown that enhanced emotional Stroop interference in highly anxious participants almost exclusively shows up as a slow sequential process affecting the trial after the presentation of an emotional stimulus, making it unlikely that attentional capture is the driving factor (cf. [57] for meta-analysis). Rather, it seems to be a process of attentional disengagement that is compromised. However, it should be noted that most evidence interpreted as evidence for pathology-related compromised attentional disengagement but unaffected attentional engagement are based on tasks requiring shifts of spatial attention that may have been affected by a process of behavioral freezing, intervening the engagement and subsequent disengagement of spatial attention, which could have been mistakenly attributed to delayed disengagement [58]. This would mean that what is interpreted as delayed attentional disengagement is ultimately due to reduced time-on-task (which is interrupted by transient ‘off-task states’), that is, reduced task engagement.

An especially attractive feature of the clinical use of conflict tasks is that they, if properly designed, reflect recovery due to therapy [59]. Apart from obvious practical benefits, studies examining changes in conflict processing induced by therapy promise to shed light on the mechanisms that underly the pathology-induced changes of performance in conflict tasks exhibited by clinical populations, including information regarding promising target mechanisms for therapeutic interventions.

Decrements in performing conflict tasks in clinical populations when conflicting stimulus attributes are related to patients’ pathology are usually explained by drawing on some variant of dual-process theory contrasting ‘automatic’ response tendencies with goal-directed controlled processing. It is assumed that either patients’ attention is excessively captured by the conflicting stimulus attribute, a process that is considered as largely automatic, or that patients have problems in shielding and/or disengaging from this information in a controlled manner, with the relative importance of these factors possibly differing among different pathologies. Although a lot of evidence supporting these claims has accrued, it should become clear that such dichotomies are certainly an oversimplification. Nevertheless, in clinical praxis such shorthand accounts may suffice as long as their limitations are kept in mind.

However, there are other possibly important factors that seem to have been largely neglected. For example, stimuli associated with patients’ personal concerns can be assumed to have higher subjective frequency, a factor that should facilitate the retrieval of associated information [60], thereby increasing the level of informational conflict. However, psychotherapy usually goes along with engaging in activities related to the respective pathology, which should rather increase than decrease the frequency of exposure to pathology-related information [59], speaking against the assumption that differences in subjective frequency alone are a main driving factor. Nevertheless, when comparing a clinical with a control group, the selection of stimulus material should carefully avoid an uneven balancing of the subjective frequency of stimuli across the two groups [61]. Similarly, it is important to consider the possibility that the valence of stimuli (e.g., their aversiveness) differs between the groups from the outset to avoid the danger of confounding stimulus valence and relevance to personal concerns [61].

The latter point may be especially relevant with respect to subjective aversiveness. First, it seems that the default response tendency when faced with aversive stimuli is avoidance [62]. Therefore, when stimuli differ in their subjective aversiveness between a clinical and a control group, this may induce implicit response conflicts to different degrees. Furthermore, recent research suggests that conflict by itself generates an aversive affective signal (cf. [63] for a review). In general, this aversive signal is assumed to motivate the engagement of cognitive control processes. However, because engaging in cognitive control is experienced as effortful and expenditure of effort is also experienced as aversive, the resource conservation principle [64] predicts that people’s readiness to invest effort should drop if they consider the task as exceedingly difficult. According to the implicit-affect-primes-effort model [65], cues associated with failure should increase the subjective difficulty of a task. For patients suffering from a specific pathology, stimuli related to this pathology can be expected to be strongly associated with failure—otherwise, they would not suffer from the pathology anymore. Therefore, it is highly plausible to assume that patients’ motivation to invest effort into a task that requires the cognitive control (e.g., inhibition) of conflicting information associated with their pathology is selectively impaired [66].

Such a view may also be able to account for a seemingly counterintuitive observation reported by [67]. These authors led snake-phobic participants perform a Stroop task with snake-related and neutral words. Participants performed this task either under standard conditions or in the presence of a boa constrictor. One might expect that the presence of the snake added to the phobics anxiety, making them more vulnerable to interference by snake-related words. What happened, however, was that participants exhibited interference by snake-related words only in the absence but not in the presence of the snake. Furthermore, they responded generally faster with the snake present. [59] explain this by assuming that participants increased their effort under conditions of real threat, which also directed subjects’ priorities away from the comparatively mildly aversive snake-related words. A closer look at the procedure that ([67], Exp. 1) applied in their snake-present condition gives rise to a slightly different interpretation: participants were first exposed to a glass tank containing the snake and then told that after performing the Stroop task that, “they would be asked to do a behavioral test to determine how close they could come to touching the snake.

Subjects were assured that they could discontinue the behavioral test at any time” ([67] p. 521). Thus, this procedure emphasized the autonomy of the participant and probably also gave the color-naming task the character of preparation for a more challenging situation, that is, it became an instrumental act for the achievement of another goal. At the very least, these findings suggest that cognitive control deficits in certain forms of psychopathology are not stable but critically depend on the amount of effort subjects are willing to mobilize in the face of stimuli that are strongly associated with experiences of failure. Along this line of reasoning, the observation that pathology-related asymmetries (as compared to control groups) often ameliorated in the course of psychotherapy, may at least in part reflect the accumulation of experiences associating pathology-related stimuli with success that gradually outweigh their associations with failure.

Conceptually, these speculations are grounded on the concept of ‘task space’, which proposes that the performance of a task is not only determined by task-relevant stimulus features as conveyed by instruction (the ‘task set’) but also by the (accidental or intended) activation of information associated with the instructed stimulus features [68,69]. These may differ quite a lot among different people and psychological conditions, which provides the rationale of employing those tasks in a clinical context)

## 5. Reflections

This article started with the proposition that the concept of conflict transforms unobservable entities into observable behavior—a form of externalization of internal ‘entities’, such as response tendencies, feelings, and attitudes. In clinical psychology, some of these are considered as inappropriate—either per se (e.g., because they are self-damaging or dangerous to others) or because they are associated with (and perhaps underlying) personal suffering (e.g., depression or anxiety). Externalization proceeds by instantiating these assumed internal entities in the form of responses to stimuli onto which these map (e.g., the word ‘fear’ maps onto anxiety). However, one may also look at this from a reverse perspective: conflict may be considered as arising from suboptimal internalization of self-regulatory demands imposed by the social environment. According to self-determination theory [70], internalization can occur in two ways, either by introjection or integration [71]. Introjection results in “a rule for action that is enforced by sanctions such as threats of guilt or promises of self-approval” ([71], p. 121). Consider, for example, someone who finds himself in a depressed state thinking that he has no reason to be sad and ought not to be so, which may result in an experience of guilt that intensifies his depressed mood. The other form of internalization postulated by self-determination theory is integration, which means that a “person identifies with a value of an activity and accepts full responsibility for doing it” (ibd.).

Drawing on self-determination theory, [72] synthesized research on the positive and negative effects of social relationships on depressed individuals. They suggest that when depressives fear that their symptoms endanger the fulfillment of the basic need of social relatedness, they actively generate interpersonal stress by exhibiting excessive reassurance seeking, which in turn increases their suffering. This can be seen as an example of maladaptive introjection of the demand of ‘feeling good’ that ultimately leads to a vicious cycle. In contrast, when a social relationship is experienced as supporting the fulfillment of the basic needs of autonomy and competence and deals with the symptoms of depression, this supports an integrative commitment to foster one’s emotion-regulation competence.

It was perhaps along these lines that the snake phobics in the snake-present condition of ([67], Exp. 1) managed to adapt to the conflict induced by snake-related words in a proactive manner. Transforming the performance of the Stroop task into an instrumental act of preparing for a much more challenging situation probably made this task comparatively easy, preventing it from being considered as excessively difficult, which would have resulted in task disengagement [65]. Furthermore, the instrumental character of the task may have induced participants to consider it as an opportunity to fulfill their need for competence in a self-determined (autonomous) manner. Future research may show inasmuch these speculations can be supported by empirical observations.

## Data Availability

This article reports no original data.

## References

[1] Ach N., Abderhalden E. (1935). Analyse des Willens. Handbuch der Biologischen Arbeitsmethoden.

[2] Russell R.W. (1950). The comparative study of conflict and experimental neurosis. Br. J. Psychol..

[3] Lewin K. (1938). The Conceptual Representation and the Measurement of Psychological Forces.

[4] Miller N.E., Hunt J.M. (1944). Experimental studies of conflict. Personality and the Behavior Disorders.

[5] Gray J.A. (1982). The Neuropsychology of Anxiety: An Enquiry into the Functions of the Septohippocampal System.

[6] Bach D.R. (2017). The cognitive architecture of anxiety-like behavioral inhibition. J. Exp. Psychol. Hum. Percept. Perform..

[7] Bugg J.M. (2008). Opposing influences on conflict-driven adaptation in the Eriksen flanker task. Mem. Cogn..

[8] Kornblum S., Hasbroucq T., Osman A. (1990). Dimensional overlap: Cognitive basis for stimulus-response compatibility–A model and taxonomy. Psychol. Rev..

[9] De Houwer J. (2001). A structural and process analysis of the Implicit Association Test. J. Exp. Soc. Psychol..

[10] Eder A.B., Leuthold H., Rothermund K., Schweinberger S.R. (2012). Automatic response activation in sequential affective priming: An ERP study. Soc. Cogn. Affect. Neurosci..

[11] De Houwer J. (2003). The extrinsic affective Simon task. Exp. Psychol..

[12] Roefs A., Huijding J., Smulders F.T.Y., MacLeod C.M., de Jong P.J., Wiers R.W., Jansen A.T.M. (2011). Implicit measures of association in psychopathology research. Psychol. Bull..

[13] Stroop J.R. (1935). Studies of interference in serial verbal reactions. J. Exp. Psychol..

[14] Cattell J.M. (1886). The time it takes to see and name objects. Mind.

[15] Brown W. (1915). Practice in associating color-names with colors. Psychol. Rev..

[16] MacLeod C.M. (1992). The Stroop task: The “gold standard” of attentional measures. J. Exp. Psychol. Gen..

[17] Logan G.D., Cowan W.B. (1984). On the ability to inhibit thought and action: A theory of an act of control. Psychol. Rev..

[18] Augustinova M., Parris B.A., Ferrand L. (2019). The loci of Stroop interference and facilitation effects with manual and vocal responses. Front. Psychol..

[19] Braver T.S. (2012). The variable nature of cognitive control: A dual mechanisms framework. Trends Cogn. Sci..

[20] Eriksen B.A., Eriksen C.W. (1974). Effects of noise letters upon the identification of a target letter in a nonsearch task. Percept. Psychophys..

[21] LaBerge D. (1983). Spatial extent of attention to letters and words. J. Exp. Psychol. Hum. Percept. Perform..

[22] Gratton G., Coles M.G., Sirevaag E.J., Eriksen C.W., Donchin E. (1988). Pre-and poststimulus activation of response channels: A psychophysiological analysis. J. Exp. Psychol. Hum. Percept. Perform..

[23] Lichtenstein-Vidne L., Henik A., Safadi Z. (2012). Task relevance modulates processing of distracting emotional stimuli. Cogn. Emot..

[24] Vince M.A. (1948). The intermittency of control movements and the psychological refractory period. Br. J. Psychol. Gen. Sect..

[25] Lappin J.S., Eriksen C.W. (1966). Use of a delayed signal to stop a visual reaction-time response. J. Exp. Psychol..

[26] Verbruggen F., Aron A.R., Band G.P., Beste C., Bissett P.G., Brockett A.T., Brown J.W., Chamberlain S.R., Chambers H.C., Colzato L.S. (2019). A consensus guide to capturing the ability to inhibit actions and impulsive behaviors in the stop-signal task. eLife.

[27] Li C.S.R., Krystal J.H., Mathalon D.H. (2005). Fore-period effect and stop-signal reaction time. Exp. Brain Res..

[28] Posner M.I., Snyder C.R.R., Solso R.L. (1975). Attention and Cognitive Control. Information Processing and Cognition.

[29] Shiffrin R.M., Schneider W. (1977). Controlled and automatic human information processing: II. Perceptual learning, automatic attending and a general theory. Psychol. Rev..

[30] Bargh J.A. (1992). The ecology of automaticity: Toward establishing the conditions needed to produce automatic processing effects. Am. J. Psychol..

[31] Hommel B., Monsell S., Driver J. (2000). The Prepared Reflex: Automaticity and Control in Stimulus-Response Translation. Control of Cognitive Processes: Attention and Performance XVIII, 18.

[32] Simon J.R. (1969). Reactions toward the source of stimulation. J. Exp. Psychol..

[33] Bertelson P. (1963). SR relationships and reaction times to new versus repeated signals in a serial task. J. Exp. Psychol..

[34] Rabbit P.M.A., Vyas S.M., Kornblum S. (1973). What is repeated in the “repetition effect”. Attention and Performance IV.

[35] Shaffer L.H. (1965). Choice reaction with variable SR mapping. J. Exp. Psychol..

[36] Williams J.A. (1966). Sequential effects in disjunctive reaction time: Implications for decision models. J. Exp. Psychol..

[37] Broadbent D.E., Meyer E.D., Kornblum S. (1993). A word before leaving. Attention and Performance XIV (silver Jubilee Volume). Synergies in Experimental Psychology, Artificial Intelligence, and Cognitive Neuroscience.

[38] Kiesel A., Steinhauser M., Wendt M., Falkenstein M., Jost K., Philipp A.M., Koch I. (2010). Control and interference in task switching—A review. Psychol. Bull..

[39] Grant D.A., Berg E. (1948). A behavioral analysis of degree of reinforcement and ease of shifting to new responses in a Weigl-type card-sorting problem. J. Exp. Psychol..

[40] Gratton G., Coles M.G., Donchin E. (1992). Optimizing the use of information: Strategic control of activation of responses. J. Exp. Psychol. Gen..

[41] Egner T. (2007). Congruency sequence effects and cognitive control. Cogn. Affect. Behav. Neurosci..

[42] Jeong H.J., Cho Y.S. (2021). The effects of induced and trait anxiety on the sequential modulation of emotional conflict. Psychol. Res..

[43] Abrahamse E., Ruitenberg M., Boddewyn S., Oreel E., de Schryver M., Morrens M., van Dijck J.P. (2017). Conflict adaptation in patients diagnosed with schizophrenia. Psychiatry Res..

[44] Saunders B., Jentzsch I. (2014). Reactive and proactive control adjustments under increased depressive symptoms: Insights from the classic and emotional-face Stroop task. Q. J. Exp. Psychol..

[45] Schmidt J.R. (2019). Evidence against conflict monitoring and adaptation: An updated review. Psychon. Bull. Rev..

[46] Botvinick M.M., Braver T.S., Barch D.M., Carter C.S., Cohen J.D. (2001). Conflict monitoring and cognitive control. Psychol. Rev..

[47] McKenna F.P., Sharma D. (2004). Reversing the emotional Stroop effect reveals that it is not what it seems: The role of fast and slow components. J. Exp. Psychol. Learn. Mem. Cogn..

[48] Joyal M., Wensing T., Levasseur-Moreau J., Leblond J., TSack A., Fecteau S. (2019). Characterizing emotional Stroop interference in posttraumatic stress disorder, major depression and anxiety disorders: A systematic review and meta-analysis. PLoS ONE.

[49] Johansson L., Ghaderi A., Andersson G. (2005). Stroop interference for food-and body-related words: A meta-analysis. Eat. Behav..

[50] Dreier M.J., Wang S.B., Nock M.K., Hooley J.M. (2021). Attentional biases towards food and body stimuli among individuals with disordered eating versus food allergies. J. Behav. Ther. Exp. Psychiatry.

[51] Epp A.M., Dobson K.S., Dozois D.J., Frewen P.A. (2012). A systematic meta-analysis of the Stroop task in depression. Clin. Psychol. Rev..

[52] Loeffler L.A.K., Satterthwaite T.D., Habel U., Schneider F., Radke S., Derntl B. (2019). Attention control and its emotion-specific association with cognitive emotion regulation in depression. Brain Imaging Behav..

[53] Kalanthroff E., Henik A., Derakshan N., Usher M. (2016). Anxiety, emotional distraction, and attentional control in the Stroop task. Emotion.

[54] Gole M., Köchel A., Schäfer A., Schienle A. (2012). Threat engagement, disengagement, and sensitivity bias in worry-prone individuals as measured by an emotional go/no-go task. J. Behav. Ther. Exp. Psychiatry.

[55] Pe M.L., Vandekerckhove J., Kuppens P. (2013). A diffusion model account of the relationship between the emotional flanker task and rumination and depression. Emotion.

[56] Khosravi P., Parker A.J., Shuback A.T., Adleman N.E. (2020). Attention control ability, mood state, and emotional regulation ability partially affect executive control of attention on task-irrelevant emotional stimuli. Acta Psychol..

[57] Phaf R.H., Kan K.J. (2007). The automaticity of emotional Stroop: A meta-analysis. J. Behav. Ther. Exp. Psychiatry.

[58] Clarke P.J., MacLeod C., Guastella A.J. (2013). Assessing the role of spatial engagement and disengagement of attention in anxiety-linked attentional bias: A critique of current paradigms and suggestions for future research directions. Anxiety Stress Coping.

[59] Williams J.M.G., Mathews A., MacLeod C. (1996). The emotional Stroop task and psychopathology. Psychol. Bull..

[60] Logan G.D. (1988). Toward an instance theory of automatization. Psychol. Rev..

[61] MacLeod C., Wenzel A., Rubin D.C. (2005). The Stroop Task in Clinical Research. Cognitive Methods and Their Application to Clinical Research.

[62] Schouppe N., De Houwer J., Richard Ridderinkhof K., Notebaert W. (2012). Conflict: Run! Reduced Stroop interference with avoidance responses. Q. J. Exp. Psychol..

[63] Dignath D., Eder A.B., Steinhauser M., Kiesel A. (2020). Conflict monitoring and the affective-signaling hypothesis—An integrative review. Psychon. Bull. Rev..

[64] Gibson W.B. (1900). The principle of least action as a psychological principle. Mind.

[65] Gendolla G.H. (2012). Implicit affect primes effort: A theory and research on cardiovascular response. Int. J. Psychophysiol..

[66] Silvestrini N., Gendolla G.H. (2019). Affect and cognitive control: Insights from research on effort mobilization. Int. J. Psychophysiol..

[67] Mathews A., Sebastian S. (1993). Suppression of emotional Stroop effects by fear-arousal. Cogn. Emot..

[68] Kleinsorge T. (2021). Cognitive Capacity, Representation, and Instruction. Front. Psychol..

[69] Xiong A., Proctor R.W., Federmeier K.D. (2018). The role of task space in action control: Evidence from research on instructions. Psychology of Learning and Motivation.

[70] Ryan R.M., Deci E.L. (2002). Overview of self-determination theory: An organismic dialectical perspective. Handb. Self-Determ. Res..

[71] Deci E.L., Eghrari H., Patrick B.C., Leone D.R. (1994). Facilitating internalization: The self-determination theory perspective. J. Personal..

[72] Ibarra-Rovillard M.S., Kuiper N.A. (2011). Social support and social negativity findings in depression: Perceived responsiveness to basic psychological needs. Clin. Psychol. Rev..

